# Post resuscitation care of out-of-hospital cardiac arrest patients in the Nordic countries: a questionnaire study

**DOI:** 10.1186/s13049-015-0141-z

**Published:** 2015-08-22

**Authors:** Sini Saarinen, Maaret Castrén, Ilkka Virkkunen, Antti Kämäräinen

**Affiliations:** Emergency Medical Services, Department of Emergency Medicine, Helsinki University Hospital and Helsinki University, PL 281, 00029 Helsinki, Finland; Department of Emergency Medicine and Services, Helsinki University Hospital and Helsinki University, Helsinki, Finland; Department of Clinical Science and Education, Södersjukhuset, Karolinska Institutet, Stockholm, Sweden; Emergency Medical Services, Tampere University Hospital, Tampere, Finland

## Abstract

**Background:**

Aim of this study was to compare post resuscitation care of out-of-hospital cardiac arrest (OHCA) patients in Nordic (Denmark, Finland, Iceland, Norway, Sweden) intensive care units (ICUs).

**Methods:**

An online questionnaire was sent to Nordic ICUs in 2012 and was complemented by an additional one in 2014.

**Results:**

The first questionnaire was sent to 188 and the second one to 184 ICUs. Response rates were 51 % and 46 %. In 2012, 37 % of the ICUs treated all patients resuscitated from OHCA with targeted temperature management (TTM) at 33 °C. All OHCA patients admitted to the ICU were treated with TTM at 33 °C more often in Norway (69 %) compared to Finland (20 %) and Sweden (25 %), p 0.02 and 0.014. In 2014, 63 % of the ICUs still use TTM at 33 °C, but 33 % use TTM at 36 °C. Early coronary angiography (CAG) and possible percutaneous coronary intervention (PCI) was routinely provided for all survivors of OHCA in 39 % of the hospitals in 2012 and in 28 % of the hospitals in 2014. Routine CAG for all actively treated victims of OHCA was performed more frequently in Sweden (51 %) and in Norway (54 %) compared to Finland (13 %), p 0.014 and 0.042.

**Conclusions:**

Since 2012, TTM at 36 °C has been implemented in some ICUs, but TTM at 33 °C is used in majority of the ICUs. TTM at 33 or 36 °C and primary CAG are not routinely provided for all OHCA survivors and the criteria for these and ICU admission are variable. Best practices as a uniform approach to the optimal care of the resuscitated patient should be sought in the Nordic Countries.

**Electronic supplementary material:**

The online version of this article (doi:10.1186/s13049-015-0141-z) contains supplementary material, which is available to authorized users.

## Background

The survival rates following out-of-hospital cardiac arrest (OHCA) are globally highly variable, ranging from 2 to 11 %, Europe averaging an overall survival of 9 % [1, 2]. These dismal figures persist despite advances in resuscitation medicine- although some centers have been able to increase survival using a goal-directed post resuscitation protocol including early routine coronary angiography (CAG) with possible percutaneous coronary intervention (PCI) and targeted temperature management at 33 °C (cooling patients to 32–34 °C for 12-24 h; also called mild therapeutic hypothermia) [3].

Evidence of optimal post resuscitation treatment is still inconclusive. Efficacy of TTM at 33 °C has been established in randomized controlled trials (RCTs) [4, 5], but Nielsen et al. showed in a large study 2013 that TTM at 33 °C had no benefit over TTM at 36 °C [6]. Also regarding the role of primary CAG with possible PCI – as concluded by Kagawa and colleagues in their review - the treatment may improve outcomes of OHCA patients, but current evidence is inconclusive [7]. Early CAG is often provided for OHCA patients with ST-segment elevation myocardial infarction (STEMI), but remains debatable whether it should be performed also to OHCA survivors with non-STEMI. Approximately every fourth patient with non-STEMI has been shown to have a culprit lesion and an indication for PCI after OHCA [8].

Although international guidelines are set to guide the treatment of cardiac arrest victims, it is likely that hospital policies regarding post resuscitation treatment vary. This may indeed be a reflection of the inconclusive nature of scientific evidence upon the optimal treatment during the post resuscitation phase, but also potentially results in variable survival rates of OHCA victims. In particular, on a national level in clinical work, we have observed that hospital policies and criteria for intensive care unit (ICU) admission of OHCA survivors vary, as well as treatment modalities provided, which might affect survival. To our knowledge, no comparison of provided post resuscitation care between the ICUs in the Nordic countries (Denmark, Finland, Iceland, Norway, Sweden) has been done. With this survey, our aim was to investigate ICU treatment and possible differences in the treatment of OHCA patients in the Nordic countries.

## Methods

First online questionnaire [see online questionnaire, Additional file 1] was created including 23 questions concerning post resuscitation care in the ICUs. Email addresses were obtained from national intensive care societies and the questionnaire was sent to the chief physicians of ICUs in Denmark, Finland, Iceland, Norway and Sweden. The first questionnaire was sent in November 2012 and a reminder letter was sent 10 days after and followed by a second one 5 weeks later.

The second questionnaire was not planned in the beginning of the study, but the publication of two large studies during 2013 and 2014 raised the idea of the additional questionnaire to investigate whether clinical practices had changed as a consequence of these new findings. Nielsen et al. [6] showed no benefit of TTM at 33 °C over TTM at 36 °C in the treatment of comatose survivors after cardiac arrest. More recently, Kim et al. reported no benefit of pre-hospital induction of TTM with cold fluids but significantly more serious adverse events in the group receiving cold fluids [9]. Second questionnaire [see online questionnaire, Additional file 2] regarding changes in clinical practices – specifically concerning TTM and PCI - during the last two years was sent to same respondents in October 2014 and a reminder one week later. Responses were collected using a web-based survey tool (surveymonkey.com).

Percentages reported are proportions of responses and Fisher's exact test was used to count p-values for categorical data. Statistical significance was set at *p* <0.05.

## Results

### First questionnaire 2012

#### Respondents and hospitals

The first questionnaire was sent to 188 ICUs, of which 84 were located in Sweden, 52 in Denmark, 28 in Norway, 22 in Finland and 2 in Iceland. Responses were received from 96 (51 %) ICUs and these were included in the study. Response rates were highest from Iceland (100 %) and Finland (72 %), followed by Sweden (48 %), Denmark (48 %) and Norway (46 %). To a single question, the response rates varied between 61 to 100 %. The responses were mainly provided by chief physicians (*n* = 67 70 %) and consultants (*n* = 25 26 %).

Of the respondents, 60 (66 %) worked in a hospital receiving all OHCA patients from that area, while 30 (34 %) reported several receiving hospitals in their catchment area. The estimated number of OHCA patients treated per year varied from zero to over forty, most hospitals treating 5–20 patients per year (38 %) or over 40 patients per year (31 %).

#### Which OHCA patients are admitted to the ICU?

Fifty one (59 %) ICUs have a predefined protocol according to which OHCA patients are admitted to the ICU. Of those ICUs having predefined criteria, 25 % stated that the time delay from the onset of the arrest to return of spontaneous circulation (ROSC) is a criterion. The maximum acceptable time from collapse to ROSC for to admit an OHCA patient to the ICU according to predefined criteria varied between 15 to 60 min, 60 min being the most common. Patients presenting with a delay from the onset of arrest to ROSC exceeding 30 min are admitted in 70 (89 %) of the ICUs.

Initial rhythm is included by 27 % in the predefined criteria for ICU admission; half of them accept only patients resuscitated from ventricular fibrillation (VF) or pulseless ventricular tachycardia (VT). In 13 ICUs (16 %) patients with initial rhythm of pulseless electrical activity (PEA) or asystole (ASY) are not admitted to the ICU. Four (5 %) ICUs responded that patients with PEA are not admitted to the ICU even if they are expected to recover and three ICUs (4 %) do not treat patients resuscitated from ASY despite chances to recover would exist.

Age is used as a predefined criterion in 16 % of the ICUs, with maximum age enabling ICU admission ranging from 65 to 80 years. Patients requiring help in activities of daily life are accepted to the ICU in 70 (89 %) of responding hospitals. Four (5 %) ICUs reported that patients with a life time expectancy under 5 years are not admitted to the ICU.

Other predefined criteria to admit a patient to the ICU were a cardiac cause for OHCA, a delay to ROSC over 15 min and Glasgow Coma Scale (GCS) score <9 [10], expectation of recovery, good or moderate quality of life before OHCA, pre-arrest health state corresponding to the American Society of Anesthesiologists (ASA) classification score 1–3 [11], witnessed onset of arrest, lack of terminal illnesses, intubation and eligibility for ICU treatment before OHCA.

#### ICU treatment

In 2012, twenty nine (37 %) ICUs treated all patients resuscitated from OHCA with TTM at 33 °C, assuming there were resources available at the moment. Reasons not to treat a patient with TTM at 33 °C included severe co-morbidities, a non-cardiac cause of OHCA or return of consciousness after resuscitation. Thirty seven percent (28) of clinicians felt comfortable treating OHCA patients without TTM, while 63 % (48) felt uncomfortable.

TTM is induced in the pre-hospital setting of 28 (35 %) ICUs. Pre-hospital TTM is induced with cold intravenous (IV) fluids, ice packs, nasal cannula, by exposing the patient to cold environment or with different combinations of the above. Cold fluids or ice packs were the most frequently used methods.

Early CAG with possible PCI was routinely provided for all survivors of OHCA in 34 (39 %) hospitals in 2012. The proportion of ICUs having a cardiac catheterization laboratory within the same facility is not known, which might affect diagnostic practices.

#### Prognostic decisions

Prognostic evaluation is mostly (38, 49 %) made >72 h after OHCA, but also earlier: between 48 to 72 h (21, 27 %), 24 to 48 h (16, 21 %) or < 24 h (3, 4 %). Prognostic evaluation is mainly based on clinical signs (70, 92 %) and EEG (50, 66 %), whereas somatosensory evoked potentials (SEP) are less used as a prognostic tool (26, 34 %). Biochemical markers such as neurone specific enolase (NSE) or protein S-100 are seldom used (NSE *n* = 7, 9 %; S-100 *n* = 2, 3 %). Magnetic resonance imaging (MRI) and computed tomography (CT) scan are also used for prognostic evaluation.

#### Regional differences in post resuscitation care

CAG - and PCI if indicated - is routinely provided for all survivors of OHCA significantly more frequently in Sweden (18, 51 %) and in Norway (7, 54 %) compared to Finland (2, 13 %), p 0.014 and 0.042 respectively. In Denmark, CAG is performed routinely in 6 (27 %) ICUs and in Iceland in 1 (50 %) ICU.

A predefined protocol was reported to exist more often in Norway (10, 77 %) and in Denmark (16, 76 %) than in Sweden (17, 49 %) or Finland (7, 47 %), NS.

TTM is induced prior to hospital admission significantly more often in Norway (9, 69 %) and in Finland (8, 53 %) compared to Sweden (4, 14 %), p 0.001 and p 0.009. In Denmark, 6 (29 %) ICUs reported TTM is induced prior to hospital admission and 1 (50 %) in Iceland (Table 1). The method for pre-hospital induction of TTM is typically the infusion of cold IV fluids in Finland and in Iceland, a nasal cooling device in Sweden, icepacks and environmental exposure in Norway and the combination of cold packs, cold IV-fluids and environmental exposure in Denmark.

**Table 1:** Number of hospitals providing targeted temperature management at 33 °C/coronary angiography for all out-of-hospital cardiac arrest survivors 2012

Country	TTM at 33 °C	Early CAG
Denmark	21 (43 %)	6 (27 %)
Finland	3 (20 %)	2 (13 %)
Iceland	1 (50 %)	1 (50 %)
Norway	9 (69 %)	7 (54 %)
Sweden	7 (25 %)	18 (51 %)

TTM = targeted temperature management

CAG = coronary angiography

All OHCA patients admitted to the ICU are treated with TTM at 33 °C significantly more often in Norway (9, 69 %) compared to Finland (3, 20 %) and Sweden (7, 25 %), p 0.02 and 0.014. Approximately half of the ICUs in Denmark (9, 43 %) and in Iceland (1, 50 %) treat all patients with TTM at 33 °C (Table 1).

### Second questionnaire on policy change 2014

The second questionnaire was sent successfully to 184 ICUs and 84 of them responded (46 %).

In 2014, 51 (61 %) respondents report that hospital policies on TTM have changed since 2012. Most (52, 63 %) ICUs still use TTM at 33 °C, but 27 (33 %) use TTM at 36 °C.

In 2014, 23 (28 %) ICUs provide routine CAG to all actively treated victims of OHCA. Most (75, 90 %) ICUs report that they have not changed policies regarding CAG and PCI during the last two years.

## Discussion

In this study, the post resuscitation care policies in the ICUs of the Nordic countries were surveyed. With a response rate of 51 %, three main findings were discovered.

First, the criteria for ICU admission are highly variable regarding the effect of the age of the patient, the initial rhythm, and the delay to ROSC. Age may be a contributing factor to poor survival from OHCA, but does not categorically define ICU care as futile [12, 13]. A non-shockable initial rhythm is associated with worse survival than a shockable initial rhythm, but again, does not indicate complete futility *per se* [5, 14]. An interesting result was that most ICUs apply active care in patients with a delay to ROSC of up to 60 min. This is in contrast with the results of Oddo et al. [5] and Väyrynen et al. [15] – in both these studies survival was unlikely with delays to ROSC exceeding 25 min. Future studies are needed to clarify the impact of different ICUs’ admission criteria on survival.

Secondly, post resuscitation care includes TTM as a routine treatment modality for all OHCA survivors only in 37 % of the ICUs, despite Scandinavian and ERC guidelines promoting this treatment also in cases of a non-shockable initial rhythm when active treatment is decided [16, 17]. Implementation of TTM has varied widely locally as well as between countries: in 2012, 69 % of ICUs in Norway reported to use TTM at 33 °C for all OHCA patients compared to 20 % in Finland. This is in line with a Norwegian study reporting that 70 % of OHCA patients received TTM at 33 °C 2004–2008 [18]. The Norwegian investigators noted that TTM was underused in older age and female subgroups similar to our results as age was a common reason to withhold ICU treatment or TTM [18]. The publication of new data upon TTM after 2012 is reflected in the practices of the responding ICUs – TTM at 36 °C has been implemented in 33 % of the responding ICUs. Still, 63 % of the responding ICUs provide TTM at 33 °C, reflecting the ongoing debate upon the evidence base of TTM at 36 °C after OHCA. Latest meta-analysis of RCTs concerning TTM revealed that TTM at 33 °C did not improve survival compared to TTM at 36 °C and both TTM at 33 and 36 °C improved survival compared to no TTM [19].

Thirty nine percent of ICUs provide CAG routinely for all survivors of OHCA. According to a recently published systematic review and meta-analysis, 7 out of 32 non-randomised studies supported early CAG with possible PCI and a pooled unadjusted odds ratio for survival of 2.78 favouring acute angiography was reported [20]. PCI has been observed to be associated with significantly higher survival after adjustment for confounders [21, 22], and also associates with better long-term functional outcome and quality of life [23]. The European Resuscitation Council guidelines 2010 recommend that angiography and PCI, if necessary, should be considered in resuscitated patients with ST-elevation myocardial infarction or new left bundle branch block and may be reasonable also in selected patients without ECG changes or prior clinical findings such as chest pain, and that angiography could be included in a standardised post-cardiac arrest protocol [24]. However, after publication of the current resuscitation guidelines, the debate upon the actual benefit of early CAG is ongoing and without conclusive evidence to support routine application [25] –in the follow up survey no significant changes to CAG policies were reported.

Thirdly, prognostic evaluation is performed mainly based on clinical signs (92 %) and in 25 % of the ICUs during 48 h following the arrest. Three ICUs report that prognostic evaluation is performed <24 h of the arrest. These results are alarming as there are no clinical neurological signs that reliably predict poor outcome <24 h after cardiac arrest, and even after 72 h following the arrest some clinical signs such as lowered GCS motor score of 2 or myoclonus are not completely reliable [17, 26, 27]. Furthermore, in association with the use of TTM at 33 °C, all prognostication should be delayed until >72 h after the initial insult, and decisions to limit or withdraw care should not rely on a single prognostication tool [17, 28].

These presented observations alongside the fact that only 59 % of the ICUs have a predefined protocol for post resuscitation care and admission criteria give rise to the assumption that post resuscitation care is not provided in a uniform fashion and according to published guidelines in the Nordic countries.

Although over half of the ICUs in the Nordic countries do have pre-set criteria defining which OHCA patients are admitted to the ICU, the criteria do not seem to present equal approaches towards post resuscitation care of the cardiac arrest survivor. Furthermore, when predefined criteria do not exist, the decision to admit the patient in the ICU is likely to be more clinician-dependent and made on a case-by-case basis.

Both of these approaches carry risks – an evidence based set of predefined criteria may prevent individual variability in terms of ICU admission assessment, but may also exclude those patients that carry potential for good outcome but do not fit these criteria. On the other hand, a case-by-case assessment allows for the evaluation of all factors associated with the initial insult, prediction of outcome and the indications for intensive care. However, individual variability in experience and opinions of the treating physician may lead to alternating patient selection even on the hospital level.

The heterogeneity in the criteria for ICU admission of the cardiac arrest survivor and thereafter provided care reflect the inconclusive evidence regarding optimal post resuscitation care. The purpose of the present study was not to evaluate whether certain treatment protocols or codes of conduct are particularly more appropriate than others, but rather to evaluate whether the intensive care treatment of cardiac arrest victims in the Nordic countries is of uniform nature. As a result, it can be speculated whether existing data on post resuscitation care are interpreted and treatment modalities implemented more locally than according to existing international guidelines. Utstein Formula for Survival Collabolators speculated that local implementation of well-documented therapies is the easiest way to reach most immediate improvement in survival [29]. It is known that several barriers to guideline implementation exist in the context of emergency medical services [30], but future studies should also take into account that implementation of best practices are truly extended throughout the complete chain of survival.

### Limitations

The main limitations of the study are the low response rates of 51 % to the first questionnaire and 46 % to the second one. It is possible that ICUs with a standardized post resuscitation protocol for OHCA patients were more willing to respond than those without a protocol, which might affect study results. However, even if a response rate of 100 % would have been received, the content of the responses would not have diminished the range of response variability and thus the differences in admission and treatment protocols. Furthermore, similar response rates are generally reported in online questionnaires. It can also be speculated whether the opinions of the individual responder could affect response contents, but the content and questions of the survey have been specifically directed at the general clinical conduct of ICUs - not at individual physicians.

## Conclusions

In the Nordic countries, criteria for OHCA patients' ICU admission are highly variable. Since 2012, TTM at 36 °C has been implemented in some ICUs, but TTM at 33 °C is used in majority of the ICUs. TTM at 33 or 36 °C and primary coronary intervention are not routinely provided for all OHCA survivors and the criteria for these treatment modalities are variable. A more uniform approach to the optimal care of the resuscitated patient should be sought in the Nordic Countries. This requires the adoption of best practices from centers with best outcomes, and higher level of evidence from clinical studies regarding optimal treatment and patient selection. Despite international guidelines, the implementation of available treatment modalities seems to be variable.

## Additional files

Additional file 1:
**Questionnaire1.pdf, is the first online questionnaire.** (PDF 115 kb) Additional file 2:
**Questionnaire2.pdf, is the second questionnaire concerning policy change.** (PDF 27 kb)
