# Supplementary material for: Post resuscitation care of out-of-hospital cardiac arrest patients in the Nordic countries: a questionnaire study
Source: Scand J Trauma Resusc Emerg Med. 2015 Aug 22;23:60. doi: 10.1186/s13049-015-0141-z (PMC4563946; doi:10.1186/s13049-015-0141-z)
Supplement: Additional file 1: — Questionnaire1.pdf, is the first online questionnaire. (PDF 115 kb) [file 13049_2015_141_MOESM1_ESM.pdf]

Survey of intensive care treatment of out-of-hospital cardiac arrest patients in tertiary hospitals in Nordic countries

1. Country

- Finland
- Sweden
- Norway
- Denmark
- Island

2. City

3. Hospital

4. Position of the answerer

- Chief physician
- Specialist doctor
- Resident doctor
- Other

5. How many out-of-hospital cardiac arrest (OHCA) patients does Your hospital treat per year?

- >40
- 21-40
- 5-20
- <5
- 0

6. Does Your hospital treat all OHCA patients treated by emergency medical service (EMS) system within the area of your hospital?

- Yes
- No

Next

Survey of intensive care treatment of out-of-hospital cardiac arrest patients in tertiary hospitals in Nordic countries

7. Number of hospitals receiving out-of-hospital cardiac arrest patients within Your area

8. Is percutaneous coronary intervention (PCI) provided routinely for all survivors of prehospital cardiac arrest?

- Yes
- No

9. Does Your intensive care unit have a predefined protocol according to which out-of-hospital cardiac arrest patients are admitted to ICU?

- Yes
- No

Previous Next

Survey of intensive care treatment of out-of-hospital cardiac arrest patients in tertiary hospitals in Nordic countries

10. If You have a predefined criteria which OHCA patients are admitted, please specify:

age maximum

initial rhythm

ROSC maximum

other

11. Are any out-of-hospital cardiac arrest patients admitted to ICU if they need help in activities of daily life?

- Yes
- No

12. Are out-of-hospital cardiac arrest patients admitted to ICU if their lifetime expectancy according to other illnesses is less than 5 years?

- Yes
- No

13. Are out-of-hospital cardiac arrest patients admitted to ICU only if they had VF/VT as initial rhythm?

- Yes
- No

14. Are out-of-hospital cardiac arrest patients with pulseless electrical activity (PEA) admitted to ICU if they are expected to recover?

- Yes
- No

15. Are out-of-hospital cardiac arrest patients with ASY admitted to ICU if they are expected to recover?

- Yes
- No

16. Are out-of-hospital cardiac arrest patients admitted to ICU if their ROSC time is >30 minutes?

- Yes
- No

17. Are all out-of-hospital cardiac arrest patients admitted to ICU treated with therapeutic hypothermia (expecting there are resources at the moment patient is admitted)?

- Yes
- No

Previous Next

Survey of intensive care treatment of out-of-hospital cardiac arrest patients in tertiary hospitals in Nordic countries

18. If not, which patients are excluded?

19. Does EMS service in Your area use induction of hypothermia in the prehospital setting?

- ○ Yes
- ○ No
- ○ I don't know

Previous Next

Survey of intensive care treatment of out-of-hospital cardiac arrest patients in tertiary hospitals in Nordic countries

20. If yes, method of induction of hypothermia (cold iv-fluids, ice packs, other...)?

21. Would You feel comfortable treating out-of-hospital cardiac arrest patients without hypothermia?

- ○ Yes
- ○ No

22. After out-of-hospital cardiac arrest, what time is the prognostic evaluation performed?

- ○ <24h
- ○ 24-48h
- ○ 48-72h
- ○ >72h

23. Is the prognostic decision based on...

- ☐ NSE laboratory test
- ☐ S-100 laboratory test
- ☐ EEG
- ☐ SEP
- ☐ clinical signs
- ☐ all of the above

other
