# Supplementary material for: Post resuscitation care of out-of-hospital cardiac arrest patients in the Nordic countries: a questionnaire study
Source: Scand J Trauma Resusc Emerg Med. 2015 Aug 22;23:60. doi: 10.1186/s13049-015-0141-z (PMC4563946; doi:10.1186/s13049-015-0141-z)
Supplement: Additional file 2: — Questionnaire2.pdf, is the second questionnaire concerning policy change. (PDF 27 kb) [file 13049_2015_141_MOESM2_ESM.pdf]

Survey of intensive care treatment of out-of-hospital cardiac arrest patients in tertiary hospitals in Nordic countries

1. Have your hospital policies on mild therapeutic hypothermia in out-of-hospital cardiac arrest patients changed since 2012?

- ( ) yes
- ( ) no

2. If active treatment of out-of-hospital cardiac arrest patient is chosen, does your intensive care unit use...

- [ ] mild therapeutic hypothermia
- [ ] only temperature control
- [ ] none of the above

3. Have your hospital policies on percutaneous coronary intervention in out-of-hospital cardiac arrest patients changed since 2012?

- [ ] yes
- [ ] no

4. Is routine percutaneous coronary intervention performed to all actively treated victims of out-of-hospital cardiac arrest?

- [ ] yes
- [ ] no

Loppu
